# Effects of attention on the control of locomotion in individuals with chronic low back pain

**DOI:** 10.1186/1743-0003-5-13

**Published:** 2008-04-25

**Authors:** Claudine JC Lamoth, John F Stins, Menno Pont, Frederick Kerckhoff, Peter J Beek

**Affiliations:** 1 Research Institute MOVE, Faculty of Human Movement Sciences, VU University Amsterdam, van der Boechorststraat 9, 1081 BT, Amsterdam, the Netherlands; 2 Rehabilitation Center Amsterdam, Department of Health and Behavior, Overtoom 283, 1054 HW, Amsterdam, the Netherlands

## Abstract

**Background:**

People who suffer from low back pain (LBP) exhibit an abnormal gait pattern, characterized by shorter stride length, greater step width, and an impaired thorax-pelvis coordination which may undermine functional walking. As a result, gait in LBP may require stronger cognitive regulation compared to pain free subjects thereby affecting the degree of automaticity of gait control. Conversely, because chronic pain has a strong attentional component, diverting attention away from the pain might facilitate a more efficient walking pattern.

**Methods:**

Twelve individuals with LBP and fourteen controls participated. Subjects walked on a treadmill at comfortable speed, under varying conditions of attentional load: (a) no secondary task, (b) naming the colors of squares on a screen, (c) naming the colors of color words ("color Stroop task"), and (d) naming the colors of words depicting motor activities. Markers were attached to the thorax, pelvis and feet. Motion was recorded using a three-camera SIMI system with a sample frequency of 100 Hz. To examine the effects of health status and attention on gait, mean and variability of stride parameters were calculated. The coordination between thoracic and pelvic rotations was quantified through the mean and variability of the relative phase between those oscillations.

**Results:**

LBP sufferers had a lower walking speed, and consequently a smaller stride length and lower mean thorax-pelvis relative phase. Stride length variability was significantly lower in the LBP group but no significant effect of attention was observed. In both groups gait adaptations were found under performance of an attention demanding task, but significantly more so in individuals with LBP as indicated by an interaction effect on relative phase variability.

**Conclusion:**

Gait in LBP sufferers was characterized by less variable upper body movements. The diminished flexibility in trunk coordination was aggravated under the influence of an attention demanding task. This provides further evidence that individuals with LBP tighten their gait control, and this suggests a stronger cognitive regulation of gait coordination in LBP. These changes in gait coordination reduce the capability to deal with unexpected perturbations, and are therefore maladaptive.

## Background

Chronic low back pain (LBP) is characterized by impaired gait, such as low walking speed, short stride length, and unflexible coordination between trunk segments [1]. It is well known that the control of healthy gait and posture [2] as well as the experience of pain, such as LBP [3-5], are under the influence of attentional factors. However, the relationship between attention and gait in LBP has seldom been addressed directly. Several theories have been formulated to explain the origin of the abnormal gait in LBP. According to one account, walkers with LBP may inadvertently adopt a strategy whereby they modify their pattern of muscular activity in an attempt to reduce the sensation of pain. In other words, they adopt a 'protective guarding' or 'splinting' strategy by restricting movements of the spine [6]. In a similar vein, the 'fear avoidance' model [7] emphasizes psychogenic factors, such as anxiety, hypervigilance and catastrophizing in the development and chronicity of musculoskeletal pain. According to this model, the enduring avoidance of physical activities that are assumed to increase pain may lead to altered gait. Finally, it has been suggested that walkers with LBP exhibit poorer motor control, and/or suffer from reduced proprioception [8,9], which limits their ability to adapt their gait pattern to changing circumstances and deal with (unexpected) perturbations. As a result, the walkers compensate for their poorer motor control by deliberately adopting a slower and less flexible gait [1]. At the very least, these accounts highlight the potential relevance of central (cognitive) factors in the regulation of gait.

One common way to study effects of cognition on gait is by examining the effect of a secondary cognitive task on the control of locomotion. The dual-task methodology has repeatedly been applied to clarify the role of attentional factors in the control of healthy and abnormal gait [10]. The picture that has emerged from these studies is that dual tasking results in gait adaptations, such as an overall lower walking speed [11] or lower step width variability [12], although the outcome is greatly affected by the type of secondary task and by subject characteristics.

The introduction of a secondary attention-demanding task with LBP sufferers may have one of two consequences. It could be the case that the prolonged experience of pain affects the degree of automaticity in the control of gait, that is, walkers with LBP coordinate their movements in a controlled (i.e. attention demanding) mode, due to poorer motor control (e.g. [1,13]). The introduction of a secondary task would then result in a temporary less flexible gait, because walkers have to actively cope with the greater information processing demands. This outcome would be consistent with the existing literature on abnormal gait in other populations. For example, it has been shown that gait of elderly individuals [14] and stroke patients [15] is affected more by an attention demanding secondary task than gait of healthy controls, as evidenced by a concomitant decrease in gait velocity. A second possibility is that a secondary task leads temporarily to a less tightly controlled gait pattern, because the task disrupts the processing of pain signals. As a result, gait can proceed in a more fluent and automatic fashion. This hypothesis is based on the notion that both acute and chronic pain have a strong attentional component, interrupting ongoing thoughts and behaviors [16,17]. For example, it has been shown that chronic LBP sufferers were able to continue a painful physical exercise for a prolonged period of time when it was combined with an attention-demanding word shadowing task [3]. Relatedly, it was found [18] that a highly attention demanding task caused a significant reduction in the experience of acute induced pain. Theoretically, diverting attention away from the sensory and affective components of pain may thus give rise to an increase in the ability to carry out certain behaviors, such as walking, in a more efficient fashion.

In the present experiment attention was manipulated using the Stroop task. A previous study showed that the Stroop task has clear effects on gait in healthy young adults, resulting in more 'conservative' gait [12], which makes the Stroop task a promising candidate to further explore the attentional demands of gait in different populations. In the present study, Stroop stimuli consisted of incongruent Stroop words (e.g., the word BLUE in a red font) which have been shown to have a clear effect on gait parameters [12]. In addition, we tested the effect of so-called movement Stroop words on gait (e.g., the word RUNNING in a yellow font). We hypothesized that these words would trigger increased attentional processing toward pain-related information in the LBP group, which would become manifest as altered gait and slower speed of naming [19].

Apart from studying more traditional gait parameters such as mean stride length, step width, and step frequency, we studied trunk coordination and the variability of trunk coordination and stride parameters. Flexible adaptations in trunk coordination to, for instance, changes in walking velocity are considered a hallmark of unaffected gait. Previous studies have shown that, contrary to unaffected gait, walkers with chronic LBP tend to perseverate in a pattern characterized by in-phase coordination between thorax and pelvis (i.e., in a pattern of coordination in which thorax and pelvis always rotate in the same direction) across walking speeds. Hence, the locomotory problems of LBP give rise to a decrease in overall gait stability [1,13]. In addition, variability of gait parameters and overall gait consistency provide important insights into the organization of healthy and pathological gait [13,20-23]. For example, rotational amplitudes of thorax and pelvis were found to be of the same magnitude in LBP sufferers and controls, whereas the coupling between the segments in the LBP group was less variable, i.e., more rigid [1,13,24]. With respect to the effect of attention on the timing of gait, healthy walkers were found to adopt a more variable gait pattern under the influence of an attention demanding dual task such as backward counting [11] and performing a verbal fluency task [25].

The objective of the present study was to elucidate the relation between attention and gait in LBP. This insight might contribute to further refining existing therapeutic schemes for the management of chronic LBP.

## Methods

### Participants

Data were collected from 12 subjects with chronic non-specific LBP (6 women, 6 men) and 14 pain free control subjects (7 women, 7 men). The mean age of the LBP group was 45 years (SD = 9.2, range 27–59), and that of the control group was 44 years (SD = 7.4, range = 28–53). This age difference was not significant. The mean length and weight of the LBP group was 174 cm (SD = 13) and 76 kg (SD = 10), respectively, and for the controls it was 176 cm (SD = 6) and 69 kg (SD = 7). The LBP participants were recruited from the outpatient department of the Rehabilitation Centre Amsterdam. All participants with LBP suffered from long lasting chronic unexplained LBP, with a duration of 7 to 15 years. Actual pain intensity during the experiment as measured with a visual analogue scale (VAS; 0 = no pain at all, 100 = severe back pain) ranged from 25 to 48.

The procedure was approved by the Ethics Committee of the Medical Centre of the VU University before the experiment was conducted. All participants gave their written informed consent to participate in the study. The inclusion criteria for the LBP participants were: (1) medical diagnosis of non-specific LBP with pain and symptoms persisting for longer than 3 months for which medical treatment had been sought, (2) age between 18 and 65 years, (3) ambulation without a walking aid, and (4) proficiency in the Dutch language. Participants were excluded if they had: (1) LBP of traumatic or structural origin, (2) LBP with neurological symptoms or pain radiation in the lower leg(s), (3) previous back surgery, (4) spinal tumors or infections, or (5) neurological and/or musculoskeletal disorders unrelated to LBP.

### Procedure

The experiment consisted of two blocks that were always performed in the same order. In the first block participant performed the conditions of the Stroop test while seated, whereas in the second block (gait block) participants performed the same Stroop conditions while walking on a treadmill for 3 minutes. The Stroop test consisted of three conditions: 1) A baseline condition (STROOP-BASE), consisting of squares that were displayed in one of four colors (yellow, blue, red, green), 2) an incongruent condition (STROOP-INCO), consisting of color words that were always shown in an incongruent font, e.g., the Dutch equivalent of the word BLUE shown in a red font, and 3) a movement Stroop condition (STROOP-MOVE), consisting of movement-related words (Dutch verbs) that were always shown in one of the four adopted font colors (Appendix 1).

The Stroop items were shown on a computer using PowerPoint. Each slide consisted of 9 Stroop items, displayed on a 3 × 3 grid. Stroop items were displayed in a large bold font, using bright colors, against a dark background. As soon as the participant had verbally labeled all 9 items on a slide the experimenter pressed a key, which triggered the appearance of the next slide. The experimenter manually scored the number of errors for each slide, while the PowerPoint software recorded the duration that each slide was shown.

In the seated block, all participants received the three Stroop conditions in the same order, starting with STROOP-BASE, which was followed by STROOP-INCO, followed by STROOP-MOVE. In each condition 11 PowerPoint slides were shown, resulting in 99 items per Stroop condition. The slides were shown on a monitor directly in front of the participant on a table. In the gait block, participants received the same three Stroop conditions, but in a random order. The stimuli were shown on a flat screen monitor positioned at eye height directly in front of the treadmill. The distance between the walker and the screen was approximately 1.5 m. These dual task conditions were always preceded by a control condition (CONTROL) during which no Stroop were shown, i.e., walking on the treadmill without performing a secondary task.

In all conditions, the participant's task was to read out loud the color of each item (squares or words) as fast as possible, regardless of the meaning of the words, and without making too many errors. For the dual-task condition, participants were instructed to neither prioritize gait nor the Stroop task, but to perform the combined task to the best of their ability (cf. [11]).

### Apparatus

Participants walked on a motorized treadmill (Biometrix™, width = 0.6 m, length 1.6 m).

Prior to testing, each participant performed a standardized 10-meter timed walking test to determine comfortable overground walking speed. Next, participants walked for 5 minutes on the treadmill, during which speed was gradually increased from 70% to 115% of the comfortable overground walking speed and then back again to 70%. Participants than had to verbally report which treadmill speed was their preferred speed. During the actual experiment, the speed of the treadmill was set to 110% of each participant's preferred speed, and the same constant speed was used for all conditions. We chose to impose a walking speed that was close to the comfortable walking speed because maintaining a speed significantly different from the preferred speed is more energy demanding than walking at a spontaneously adopted speed [20], which could interfere with the attentional demands of the secondary task. All participants wore a safety belt while walking on the treadmill that was attached to the ceiling, but did not interfere with movements of the trunk or limbs. Participants were instructed to walk as naturally as possible in the middle of the belt, without holding or touching the handrail.

Movements were recorded using a 3D passive marker movement registration system (Simi Reality Motion System; SIMI). Three cameras recorded the movements; two were placed laterally to and slightly behind the treadmill and one camera was placed directly behind the treadmill. Six small light reflective markers were attached to the walker's body as follows: Two markers were attached to the lateral malleolus with a thin neoprene strip. Motions of these markers were used to calculate the stride parameters. Two additional markers were attached to thin metal rods that protruded sideways from a purpose-built light-weight harness worn by each participant. These markers were placed approximately 10 cm laterally to the left and right acromion. The two remaining markers were placed at the tips of an aluminium T-frame protruding approximately 20 cm caudally at the level of the spina iliaca posterior superior from a neoprene belt that was strapped around the waist. Motions of these two sets of markers were used to calculate transverse plane movements of the thorax and pelvis, and the relative phase between the pelvic and thoracic oscillations. Movements were recorded with a sample frequency of 100 Hz. During the CONTROL and STROOP conditions participants walked for 2 minutes, after which data capturing of the markers started. Irrespective of the walking speed of the participant, for each trial a fixed number of 25 consecutive strides were recorded and analyzed off line.

### Data analysis

After digitization, for each of the six markers, the data were transformed to *xyz *cartesian coordinates, with the *x*-axis corresponding to the line of progression, the *y*-axis perpendicular to the *x*-axis and parallel to the ground, and the *z*-axis pointing vertically upward. For each trial, we first determined the moments of heel strike of each foot, based on the minima of the left and right ankle markers along the *z*-axis time series. These moments were used to calculate the duration of each step (time difference between two consecutive steps) and the duration of each stride (time difference between consecutive ipsilateral steps). Stride length was determined by multiplying stride time by the speed of the treadmill, and by then adding the (positive or negative) change in the *x*-direction of the marker at the moment of heel strike relative to the position of the marker at the preceding step (e.g., [26]). Step frequency was 1/(step duration). Step width was calculated by taking the difference in the *y*-direction of each consecutive step.

Angular rotations of the pelvis and thorax were obtained form the angles of the segment with respect to the axial in the transverse plane of motion and calculated as the four quadrant arctangent, specified by the *xy*-coordinates of the two markers of the pelvis and thorax segment. The resulting time series were filtered with a second-order Butterworth zero phase forward and a reverse digital filter with a cut-off frequency of 10 Hz. From the angular rotations we derived a continuous estimate of the relative phase between pelvis and thorax in the transverse plane, following the method described in [13,24,27] with in-phase coordination denoting synchronous rotations of the segments in the same direction, and anti-phase coordination denoting synchronous rotations in the opposite direction.

### Statistical analysis

We analyzed the average time to name the 9 Stroop items on each PowerPoint slide as a function of group (LBP versus controls), activity (seated or walking) and condition (BASE, INCO, and MOVE), using a mixed-model analysis of variance (ANOVA). The difference in self-selected treadmill speed between the groups was examined using a *t*-test. The following gait parameters were analyzed: means and standard deviations (*SD*s) of stride length (cm), step frequency (Hz), step width (cm), and pelvis-thorax relative phase (deg.). These variables were analyzed with a repeated measures ANOVA with between-factor Group (LBP versus controls) and within-factor Condition (CONTROL, BASE, INCO, and MOVE). Since the *SD*s were not normally distributed, we first applied a log transformation to the variability scores before doing the ANOVA (see also [20]). To evaluate the strength of the significant effects Cohen's *f *was calculated according to: $f=\sqrt{\frac{\eta^{2}}{1-\eta^{2}}}$. An effect size (*f*) of > .4 was considered to reflect a strong effect [28]. Significant main effects were examined using post-hoc *t*-tests and using Cohen's *d *to quantify the effect size. For all tests we adopted a significance level of .05.

## Results

### Stroop performance

The ANOVA on the Stroop times revealed a main effect of group, *F*(1, 23) = 6.94, p < .05, *f *= .55, with the LBP group being overall slower than the controls (8.0 vs 6.5 s). In addition, there was an effect of Stroop condition, *F*(2, 46) = 97.94, p < .001, *f *= 2.06. Post-hoc test revealed that all three conditions differed significantly from each other (Stroop-BASE vs. Stroop-MOVE: t(24) = 3.42, p < .01, *d *= .26; Stroop-BASE vs. Stroop-INCO: t(24) = 11.18, p < .001, *d *= 1.23; Stroop-MOVE vs. Stroop-INCO: t(24) = 9.23, p < .001, *d *= 1.05), with Stroop-BASE being the fastest (6.4 s), followed by Stroop-MOVE (6.8 s), and Stroop-INCO being the slowest (8.6 s). Finally, there was a significant activity by condition interaction, *F*(2, 46) = 4.33, p < .05, *f *= .43. A post-hoc test revealed that this was due to the Stroop-INCO condition, which was performed somewhat faster during walking than while seated (t(24) = 2.15, p < .05, *d *= .23; 8.3 vs. 8.8 s, respectively). No other effects were significant.

### Gait parameters

The self-selected speed of the treadmill was higher for the controls (4.3 km/h) than for the LBP group (3.7 km/h; *t*(23) = 2.2, p < .05, *d *= .82).

**Figure 1:**
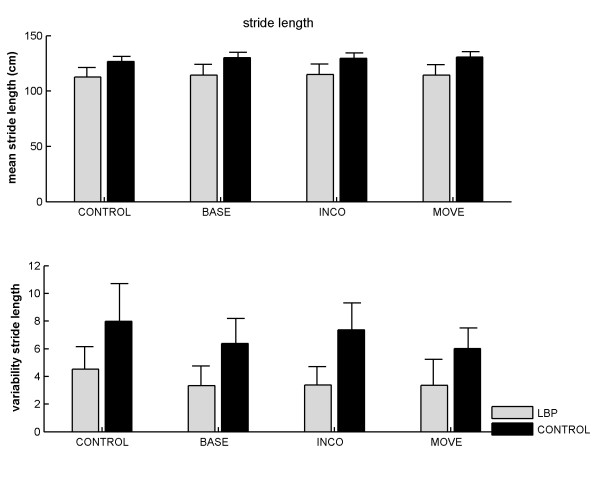
**Mean (upper panel) and variability (lower panel) of stride length as a function of group and Stroop condition.** CONTROL = walking without Stroop test; BASE = baseline Stroop condition; INCO = incongruent Stroop condition; MOVE = movement related Stroop condition. Error bars represent standard errors.

As no significant differences were found between left and right steps in both groups, we only report the results for stride length. There was a main effect of condition, *F*(3, 69) = 7.99, p < .001, *f *= .59, on stride length (Figure 1; upper panel). Post-hoc comparisons revealed that walking during the CONTROL condition (i.e., without a dual task) proceeded with shorter strides than during all other conditions (120 vs. 123 cm, respectively; CONTROL vs. BASE: *t*(24) = 3.49, p < .01, *d *= .11; CONTROL vs. INCO: *t*(24) = 3.28, p < .01, *d *= .11; CONTROL vs. MOVE: *t*(24) = 3.19, p < .01, *d *= .13). It could be that the shorter stride length in the CONTROL condition relative to the other dual-task conditions was due to some additional familiarisation of the participants with the treadmill, as this condition was always presented first. In order to test for possible sequence effects we ran an extra ANOVA with trial order (first, second, third, and fourth) as within-subjects factor, and group as between-subjects factor on the stride length scores. Again, we found that the first condition (which was thus the CONTROL condition) was significantly faster than the second, third, and fourth condition (*F*(3, 69) = 8.11, p < .001; 120.5 vs. 123.1, 123.3, and 123.6 cm, respectively), and that none of the other contrasts was significant. In other words, no further familiarisation (if any) took place after the first condition, which renders it likely that the observed effects are due to the effects of dual-tasking and not to the order of presentation of the conditions.

The main effect of group on stride length was not significant but inspection of the data revealed that one of the control subjects walked with extremely short strides. The same analysis without this subject revealed a main effect of group, *F*(1, 22) = 4.53, p < .05, *f *= .45; LBP sufferers walked with shorter strides than the controls (114 ± 0.29 vs. 133 ± 0.16 cm, respectively). Analysis of variability of stride lengths revealed that individuals with LBP walked with a less variable gait than controls (3.6 vs. 6.9 cm, respectively), *F*(1, 23) = 10.08, p < .001, *f *= .67. No significant effect of condition was observed on stride variability (Figure 1; lower panel).

There was a significant main effect of condition on step frequency, *F*(3, 69) = 4.18, p < .01, *f *= .42. Post-hoc comparisons revealed that during the CONTROL condition participants had a higher step frequency than during all other conditions (.91 vs. .89 Hz, respectively; CONTROL vs. BASE: *t*(24) = 2.13, p < .05, *d *= .11; CONTROL vs. INCO: *t*(24) = 2.40, p < .05, *d *= .11; CONTROL vs. MOVE: *t*(24) = 2.54, p < .05, *d *= .13). Condition had no significant effect on the variability of step frequency. No significant main effect of group was observed for mean and variability of step frequency.

There were no significant effects of group and condition on the mean and variability of step width. The average step width of the LBP group and the controls was 23.5 and 22.2 cm, respectively.

#### Pelvis-thorax relative phase

**Figure 2:**
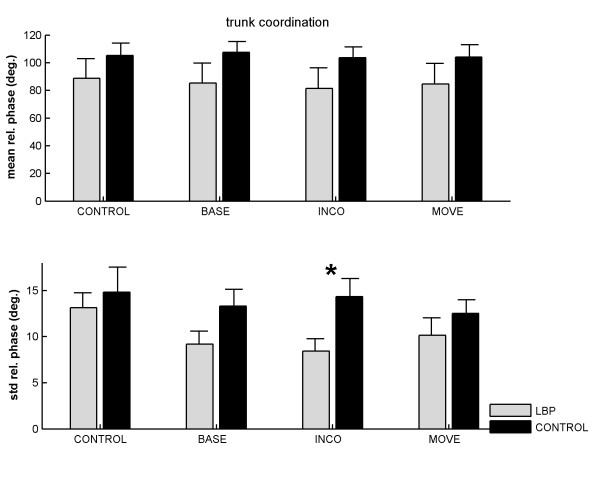
**Mean (upper panel) and variability (lower panel) of relative phase between pelvis and thorax rotations as a function of group and Stroop condition.** CONTROL = walking without Stroop test; BASE = baseline Stroop condition; INCO = incongruent Stroop condition; MOVE = movement related Stroop condition. Error bars represent standard errors. Asterisk indicates a significant (*p *< .05) difference between the two levels.

Across groups and conditions mean relative phase was smaller in the LBP group (85.05° ± 28.23°) although not significantly different from the control group (105.12° ± 46.53°) (Figure 2, upper panel). A significant main effect of condition was observed for the variability of relative phase *F*(3, 69) = 6.92, p < .001, *f *= .55, which was modified by a significant group by condition interaction, *F*(3, 69) = 3.22, p < .05, *f *= .37. The condition effect appeared to be due to the CONTROL condition, which was significantly more variable than the dual task conditions (CONTROL vs. BASE:*t*(24) = 2.94, p < .01, *d *= .45; CONTROL vs. INCO: *t*(24) = 3.01, p < .01, *d *= .46; CONTROL vs. MOVE: *t*(24) = 3.06, p < .01, *d *= .48). The interaction appeared to be due to the Stroop-INCO condition, during which LBP sufferers exhibited less variability in pelvis-thorax coordination than controls, *t*(23) = 2.77, p < .05, *d *= 1.09. The (untransformed) means for all conditions are shown in Figure 2 (lower panel).

## Discussion

The aim of this study was to clarify the role of attention in the organization of the pathologic gait observed in LBP sufferers. To this end, we compared the effect of a cognitive secondary task on a range of gait parameters in a group of LBP sufferers and a group of controls. Based on earlier studies on the control of pathologic gait we reasoned that the gait pattern in people with LBP would affect the degree of automaticity and flexibility in the control of gait, at least for the duration of the secondary task. Our results were as follows.

First, we found that, across conditions, individuals with LBP walked with a slower velocity and took shorter strides than controls. In addition, stride lengths were less variable than for the controls. These data confirm the general notion that individuals with LBP adopt a less flexible gait than controls. In addition, individuals with LBP were slower overall on the Stroop task than the controls, both seated and during locomotion. A similar finding was reported by [29], who found that chronic pain patients (mostly lower back pain patients) were slower on the color Stroop task than controls. These findings are consistent with the more general notion that cognitive abilities are impaired due to the prolonged experience of pain [30].

Second, we found that, across groups, gait was affected by the execution of the Stroop task, but that the type of Stroop task (blocks, incongruent words, or movement related words) did not seem to matter. More specifically, the Stroop task caused participants (in both groups) to adopt a gait pattern involving a lower stride frequency, accompanied by a greater stride length. Further, the Stroop task resulted in less variable pelvis-thorax coordination, although the mean phase difference between the segments remained about the same across conditions. These results suggest that the attentional demands of the task interfere with the control of locomotion (see also [12]). Interestingly, another study [31] found a complementary pattern of results: while walking on a treadmill the gait cycle was unaffected by the execution of a secondary probe RT task, but RTs were in general slower while walking than while sitting. This suggest that in a dual-task setting walkers may sometimes prioritize gait at the expense of cognitive performance (our study), and at other times cognitive performance at the expense of gait [31,32]. The factors that underlie prioritization in dual task settings are as of yet unknown. An unexpected finding was that, for both groups, the most difficult Stroop condition (INCO) was performed faster during walking than while seated. A possible explanation might be that the bodily activity (i.c., treadmill walking) caused an increase in the efficacy of prefrontal functioning, which is needed to resolve the response conflict associated with the incongruent Stroop words. For example, a recent study [33] showed that a single aerobic exercise resulted in superior performance on a test of cognitive flexibility.

Our main interest was in the possible combined (interaction) effects of attentional performance (Stroop) and gait, because these could hint at abnormal information processing in individuals with LBP. Contrary to our expectations, the movement-related Stroop words had no effect on either the Stroop naming times, nor on the control of gait. Apparently, Stroop items that were assumed to automatically 'capture' attention, due to their threat value, did not cause a processing bias. This negative finding is consistent with other studies that failed to find attentional bias in people with chronic pain using the Stroop task [19,34,35]. However, we did find that the most attention demanding task, i.e., involving naming incongruent Stroop words, had a differential effect on the LBP group as indicated by the significant group by condition interaction for the variability of relative phase. More precisely, in individuals with LBP the variability of pelvis-thorax coordination was reduced to a greater extent than in controls. Apparently, this task induced a more 'rigid' upper body coordination in the LBP group than the controls, indicating a more tightly constrained and less flexible gait. Note that although LBP participants walked slower overall, no main effect of group on the mean and variability of relative phase was observed.

From these findings it appears that gait adaptations occur under the performance of an attention demanding task, and more so in people with chronic low back pain. This notion is consistent with the idea that normal gait is to a certain extent attention demanding (e.g. [31]), and probably more so in LBP sufferers. Apparently, LBP sufferers invest cognitive (conscious) resources in the regulation of gait, and when cognitive resources are diverted to an attention demanding task, walkers reduce the complexity of maintaining their gait pattern, resulting in a reduction of gait variability. This is in line with previous studies suggesting that individuals with LBP tighten their gait control by reducing the number of degrees of freedom to cope with and hence in dealing with perturbations [1,32]. Patently, this leads them to adopt a slower and more controlled gait. Furthermore, the addition of an attention demanding task causes an aggravation of this behavior. In a sense, the secondary task can be considered a perturbation of the information processing system, which is already highly active in maintaining the abnormal gait pattern. In order to cope with the increased complexity of the dual task walkers with LBP even further reduce the flexibility and adaptability of their gait, as evidenced by more rigid upper body coordination.

## Conclusion

We found that gait in LBP sufferers is characterized by less variable upper body movements, and that the lack of flexible trunk coordination is aggravated under the influence of an attention demanding task. This finding, in combination with overall poorer performance on the cognitive task, suggests that abnormal gait is partly due to subtle disturbances in information processing that have a negative impact on both cognitive and motor performance. For clinical practice the results of the present study imply that therapeutic interventions should pay attention to movement coordination as well as cognitive abilities in the management of LBP.

## Competing interests

The authors declare that they have no competing interests.

## Authors' contributions

CJCL was the main investigator of the study, analyzed the gait data and was involved in revising the manuscript. JFS drafted the manuscript, was involved in the design of the study and in the data analysis. MP and FK recruited participants of the LBP group and were involved in the design of the study. PJB was involved in drafting and revising the manuscript.

All authors read and approved the final manuscript.

## Appendix

**Table 1:** List of movement Stroop words (Dutch original in parentheses)

walking	(lopen)
jumping	(springen)
climbing	(klimmen)
waving	(zwaaien)
kicking	(schoppen)
bending	(bukken)
lifting	(tillen)
clambering	(klauteren)
skating	(schaatsen)
playing football	(voetballen)
jogging	(joggen)
leaning	(buigen)
skiing	(skiën)
exercising	(trainen)
dancing	(dansen)
hopping	(hinkelen)
juggling	(jongleren)
swimming	(zwemmen)
sprinting	(sprinten)
